# Fractional Flow Reserve and Instantaneous Wave-Free Ratio as Predictors of the Placebo-Controlled Response to Percutaneous Coronary Intervention in Stable Coronary Artery Disease

**DOI:** 10.1161/CIRCULATIONAHA.124.072281

**Published:** 2024-10-27

**Authors:** Michael J. Foley, Christopher A. Rajkumar, Fiyyaz Ahmed-Jushuf, Florentina Simader, Shayna Chotai, Henry Seligman, Krzysztof Macierzanka, John R. Davies, Thomas R. Keeble, Peter O’Kane, Peter Haworth, Helen Routledge, Tushar Kotecha, Gerald Clesham, Rupert Williams, Jehangir Din, Sukhjinder S. Nijjer, Nick Curzen, Manas Sinha, Ricardo Petraco, James Spratt, Sayan Sen, Graham D. Cole, Frank E. Harrell Jr, James P. Howard, Darrel P. Francis, Matthew J. Shun-Shin, Rasha Al-Lamee

**Affiliations:** National Heart and Lung Institute, Imperial College London, United Kingdom (M.J.F., C.A.R., F.A.-J., F.S., S.C., H.S., K.M., S.S.N., R.P., S.S., G.D.C., J.P.H., D.P.F., M.J.S.-S., R.A.-L.).; Department of Cardiology, Imperial College Healthcare NHS Trust, London, United Kingdom (M.J.F., C.A.R., F.A.-J., F.S., S.C., H.S., S.S.N., R.P., S.S., G.D.C., J.P.H., D.P.F., M.J.S.-S., R.A.-L.).; Department of Cardiology, Essex Cardiothoracic Centre, Mid and South Essex NHS Foundation Trust, Basildon, United Kingdom (J.R.D., T.R.K., G.C.).; Medical Technology Research Centre, Anglia Ruskin School of Medicine, Chelmsford, United Kingdom (J.R.D., T.R.K.).; Department of Cardiology, University Hospitals of Dorset NHS Foundation Trust, Bournemouth, United Kingdom (P.O., J.D.).; Department of Cardiology, Portsmouth Hospitals University NHS Trust, United Kingdom (P.H.).; Department of Cardiology, Worcestershire Acute Hospitals NHS Trust, Worcester, United Kingdom (H.R.).; Department of Cardiology, Royal Free London NHS Foundation Trust, United Kingdom (T.K.).; Department of Cardiology, St George’s University of London, United Kingdom (R.W., J.S.).; Department of Cardiology, University of Southampton School of Medicine & University Hospital Southampton NHS Foundation Trust, United Kingdom (N.C.).; Department of Cardiology, Salisbury Hospital NHS Foundation Trust, United Kingdom (M.S.).; Department of Cardiology, Buckinghamshire Healthcare NHS Trust, High Wycombe, United Kingdom (R.P.).; Department of Biostatistics, Vanderbilt University Medical Centre, Nashville, TN (F.E.H.).

**Keywords:** angina, stable, cardiovascular physiology, clinical trials, randomized, coronary artery disease, ischemia, myocardial

## Abstract

**BACKGROUND:**

ORBITA-2 (the Placebo-Controlled Trial of Percutaneous Coronary Intervention for the Relief of Stable Angina) provided evidence for the role of percutaneous coronary intervention (PCI) for angina relief in stable coronary artery disease. Fractional flow reserve (FFR) and instantaneous wave-free ratio (iFR) are often used to guide PCI; however, their ability to predict placebo-controlled angina improvement is unknown.

**METHODS:**

Participants with angina, ischemia, and stable coronary artery disease were enrolled, and anti-anginal medications were stopped. Participants reported angina episodes daily for 2 weeks using the ORBITA smartphone symptom application (ORBITA-app). At the research angiogram, FFR and iFR were measured. After sedation and auditory isolation, participants were randomized to PCI or placebo before entering a 12-week blinded follow-up phase with daily angina reporting. The ability of FFR and iFR, analyzed as continuous variables, to predict the placebo-controlled effect of PCI was tested using Bayesian proportional odds modeling.

**RESULTS:**

Invasive physiology data were available for 279 patients (140 PCI and 139 placebo). The median (interquartile range) age was 65 years (59.0–70.5), and 223 (79.9%) were male. Median FFR was 0.60 (0.46–0.73), and median iFR was 0.76 (0.50–0.86). The lower the FFR or iFR, the greater the placebo-controlled improvement with PCI across all end points. There was strong evidence that a patient with an FFR at the lower quartile would have a greater placebo-controlled improvement in angina symptom score with PCI than a patient at the upper quartile (FFR, 0.46 versus 0.73: odds ratio, 2.01; 95% credible interval, 1.79–2.26; probability of interaction, >99.9%). Similarly, there was strong evidence that a patient with an iFR at the lower quartile would have greater placebo-controlled improvement in angina symptom score with PCI than a patient with an iFR at the upper quartile (iFR, 0.50 versus 0.86: odds ratio, 2.13; 95% credible interval, 1.87–2.45; probability of interaction, >99.9%). The relationship between benefit and physiology was seen in both Rose angina and Rose nonangina.

**CONCLUSIONS:**

Physiological stenosis severity, as measured by FFR and iFR, predicts placebo-controlled angina relief from PCI. Invasive coronary physiology can be used to target PCI to those patients who are most likely to experience benefit.

**REGISTRATION:**

URL: https://www.clinicaltrials.gov; Unique identifier: NCT03742050.

**Clinical Perspective**

**What Is New?**

- On little or no anti-anginal medications in ORBITA-2 (The Placebo-Controlled Trial of Percutaneous Coronary Intervention for the Relief of Stable Angina), prerandomization fractional flow reserve (FFR) and instantaneous wave-free ratio (iFR) predicted the symptom improvement with percutaneous coronary intervention (PCI) compared with placebo, as well as treadmill exercise time and stress echocardiography ischemia.
- The association between invasive physiology and outcome improvement was continuous. The lower the FFR and iFR, the greater the improvement in all outcomes with PCI.
- Patients with atypical symptoms required lower FFR and iFR values to benefit from PCI compared with patients with typical symptoms.

**What Are the Clinical Implications?**

- FFR and iFR predict the ability of PCI to improve angina symptoms under placebo-controlled conditions.
- These relationships are continuous, and the interaction is affected by symptom characteristics.
- FFR and iFR can be used as additional tools to identify patients with stable angina who are most likely to have symptom benefit with PCI.
- In patients with atypical symptoms, lower FFR and iFR values are needed to predict symptom benefit with PCI.

**Editorial, see p 215**

Percutaneous coronary intervention (PCI) relieves anatomical stenosis and ischemia in the setting of stable coronary artery disease.^1^ It has been widely used and endorsed in international guidelines for angina relief on the basis on unblinded trials and clinical experience.^2–4^ The first placebo-controlled trial of PCI, ORBITA (Objective Randomized Blinded Investigation With Optimal Medical Therapy of Angioplasty in Stable Angina, was conducted in patients with single-vessel and angiographically severe coronary artery disease who were taking the maximum tolerated anti-anginal medication.^1^ In ORBITA, 1 in 5 patients was more likely to be free from angina with PCI than placebo.^5^ However, PCI did not improve exercise time or quality of life, despite near normalization of ischemia on dobutamine stress echocardiography (DSE).^6^ Although fractional flow reserve (FFR) and instantaneous wave-free ratio (iFR) predicted the improvement in stress echocardiography ischemia with PCI compared with placebo, they did not predict the placebo-controlled impact of PCI on angina.^5^ This result was surprising and suggested that the link between stenosis, ischemia, and symptoms is complex. It is possible that this link is modified by the presence of anti-anginal medications, which have placebo-controlled evidence of symptom benefit^7–9^ and have been demonstrated to improve myocardial ischemia.^10^

ORBITA-2 (the Placebo-Controlled Trial of Percutaneous Coronary Intervention for the Relief of Stable Angina) demonstrated the placebo-controlled efficacy of PCI on symptoms in patients with single and multivessel coronary artery disease on little or no anti-anginal medication.^11^ Although, on average, PCI was more effective than placebo, 59% of patients remained symptomatic at follow-up with significant heterogeneity of treatment effect. The ORBITA-2 data allow us to explore this variability, with the aim of identifying the best predictors of treatment response to PCI.

The first secondary analysis of ORBITA-2 investigated the association between symptom characteristics and placebo-controlled response to PCI.^12^ It revealed that PCI was most likely to improve symptoms in patients who presented with typical angina. In patients with atypical symptoms, PCI had limited efficacy compared with placebo. After symptom evaluation, the next step in the clinical pathway often involves assessment of anatomy or ischemia.^4,13^ Abnormalities in these noninvasive tests often result in referral to the cardiac catheterization laboratory, where invasive physiological assessment is recommended for confirmation of ischemia before PCI.^13,14^ This secondary analysis of ORBITA-2 investigates the predictive value of FFR and iFR on the placebo-controlled efficacy of PCI.

## METHODS

The data, analytical methods, and study materials will not be made available to other researchers for the purposes of reproducing the results or replicating the procedure.

### Study Design

ORBITA-2 was a randomized, double-blind, placebo-controlled trial of PCI, conducted at 14 sites in the United Kingdom. The design has been described previously.^15^ Patients with angina and angiographically severe single and multivessel coronary artery disease on invasive coronary angiography or computed tomography coronary angiography were enrolled between November 12, 2018, and June 17, 2023. Anti-anginal medications were stopped, and patients entered a 2-week symptom assessment phase with daily reporting of angina episodes using the ORBITA smartphone symptom application (ORBITA-app).^16^ Only symptomatic patients progressed to randomization. Angina and quality of life questionnaires (Seattle Angina Questionnaire [SAQ] and EQ-5D-5L), Canadian Cardiovascular Society (CCS) class, treadmill exercise testing, and DSE were completed at prerandomization and follow-up. The typicality of angina was assessed before randomization using the Rose questionnaire. The trial was approved by the London Central Research Ethics Committee (reference 18/LO/1203). Written informed consent was obtained from each patient. Patients who did not undergo complete invasive physiological assessment were excluded from this analysis.

### Invasive Physiological Assessment

After the 2-week symptom assessment phase, patients attended for research angiography and the randomization procedure. Patients wore over-the-ear headphones playing music to establish auditory isolation. Coronary angiography was performed through the radial or femoral approach, followed by a systematic physiological investigation of every vessel that was ≥2 mm with an angiographic stenosis ≥50%. A guiding catheter was advanced to the coronary artery of interest. A total of 100 U/kg of heparin and 300 mcg of isosorbide dinitrate was administered. A pressure-tipped intracoronary wire (Verrata Wire, Philips, or Omniwire, Philips) was normalized and advanced until the pressure sensor was ≥3 vessel diameters distal to the most distal coronary stenosis, and FFR and iFR were measured. FFR measurement used peripherally administered intravenous adenosine at 140 mcg/kg per min to establish hyperemia. After completion of the measurements, the pressure wire was withdrawn, and a drift check completed. If the ratio of distal to aortic pressure in the normalization position was 1.00±0.02, the wire was renormalized, and the measurements were repeated. Participants who had no objective evidence of ischemia did not proceed to randomization. Patients with discordant invasive physiology values and no other evidence of ischemia could progress to randomization if either their FFR or iFR value was beneath the clinically used threshold. Unlike in ORBITA, the FFR and iFR values were made available to the operators. Before randomization, all stenoses that were suitable for PCI with evidence of ischemia were documented in the case report form.

### Blinding and Randomization

After physiological assessment, patients eligible for randomization received incremental doses of opiates and benzodiazepines to achieve a deep level of conscious sedation. Patients were then randomized 1:1 to PCI or placebo using open-source software.^17^ Auditory isolation was maintained throughout the procedure.

PCI was performed using standard techniques. Operators were instructed to aim for complete revascularization. Intracoronary imaging to guide and optimize the PCI was encouraged. After PCI, FFR and iFR were remeasured. Operators were permitted to use these data to guide further stent optimization and, in these cases, FFR and iFR were remeasured.

The patient, clinical staff, and research team performing follow-up assessments were all blinded to treatment allocation using the methodology described previously.^15^

### Study End Points

#### Angina Symptom Score and Daily Angina Episodes: ORBITA Smartphone Application

On enrollment to the trial, patients were taught to use a dedicated smartphone application for daily angina reporting. The design and features of the ORBITA-app have been reported previously and are included in the Supplemental Material.^16^ The angina symptom score was an ordinal clinical outcome score related to angina health status. It was calculated daily from the number of angina episodes reported by a patient on the ORBITA-app, the number of standardized units of anti-anginal medication prescribed on that day, and high-level category override events (death, myocardial infarction, and unblinding because of intolerable angina).

We additionally report the relationship between FFR and iFR on the number of angina episodes reported on the ORBITA-app in isolation, without contribution from other elements of the angina symptom score.

#### Treadmill Exercise Time

Patients underwent treadmill exercise testing at prerandomization and follow-up, using the modified Bruce protocol. Details of the treadmill exercise protocol have been reported previously.^15^

#### Patient-Reported and Physician-Assessed Symptoms and Quality of Life

Angina and quality-of-life questionnaires, including SAQ and EQ-5D-5L, were administered at prerandomization and follow-up. The SAQ consists of multiple domains including physical limitation, angina frequency, and quality of life.^18^ The EQ-5D-5L questionnaire comprises 2 distinct quality of life measures: the EQ-5D-5L descriptive system, which was combined with the United Kingdom value set producing an index value, and the EQ-5D-5L visual analogue scale (EQ VAS).^19^ Physician assessment of angina using CCS class took place before prerandomization and at follow-up.

#### Dobutamine Stress Echocardiography

DSE was performed at prerandomization and follow-up. The patient, physician, and cardiac sonographer were all blinded to treatment allocation at follow-up (Supplemental Material). Interpretation of the DSE was performed in duplicate by 3 cardiac imaging consultants. They were blinded to scan time point, their own previous assessment, and treatment allocation, using a previously reported methodology, such that each scan received 6 independent assessments.^6^

#### Contrasting the Stratified Effect Using Angina Typicality

The Rose angina questionnaire was designed as a tool to detect underlying coronary artery disease from a patient’s description of their symptoms.^20^ Chest pain or discomfort that comes on with exertion, causes them to stop, is relieved by rest within 10 minutes, and is located in the center of the chest or left chest and left arm, we designated “Rose angina.” Patients with symptoms that were felt to represent angina by the referring physician, but that fell outside any of these parameters, we designated “Rose nonangina.” The treatment effect with PCI in patients with Rose angina, stratified by FFR and iFR, was compared with the same stratified PCI treatment effect in patients with Rose nonangina.

### Statistical Analysis

In this article, the analyses as specified in the primary publication and its associated statistical analysis plan are presented, but in this physiology subset (ie, those with both FFR and iFR). The statistical approach used Bayesian ordinal regression modeling. In brief, for the primary end point, the daily angina symptom score, and the number of daily episodes of angina, the daily odds ratio (OR) of transitioning to a better angina symptom score or fewer daily episodes of angina was calculated. ORs were constructed such that an OR >1 reflected a reduction in the number of episodes of angina. The OR was derived by constructing a Bayesian Markov longitudinal ordinal model. This model included the value from the previous day (a first-order Markov model), the mean score or daily angina episodes during the prerandomization period, trial day number, and randomization allocation. Trial day number was allowed to interact with the randomization allocation to allow the model to detect variation in the treatment effect over time. The effects were allowed to be nonlinear with restricted cubic splines, and partial proportional odds with constraints. Bayesian ordinal regression models were constructed for the secondary end points, measured at prerandomization and follow-up. In these models, the follow-up value was conditioned on the prerandomization value and allowed to interact with the randomization allocation. Nonlinearity was allowed with the use of a restricted cubic spline with 3 knots on continuous predictors.

To assess the impact of FFR and iFR on the placebo-controlled effect of PCI, these variables were included in these original models and allowed to interact with the randomization arm. Nonlinearity was allowed with the use of a restricted cubic spline with 3 knots. For each patient, the single lowest FFR or iFR value was used.

To assess the impact of symptoms characteristics, a variable indicating whether the patient had Rose angina or Rose nonangina was also introduced and allowed to interact with the randomization arm and baseline physiology.

To test whether FFR or iFR affected the response to PCI, patients at the lower and upper quartiles of FFR and iFR values were compared, and the probability that more severe physiology derives greater benefit than less severe physiology was tested. A similar approach was used to compare the impact in patients with Rose angina and nonangina.

## RESULTS

ORBITA-2 enrolled 439 patients, of whom 301 were randomized; 151 were allocated to PCI and 150 were allocated to placebo. Eleven patients in the PCI group and 11 patients in the placebo group did not undergo invasive physiological assessment. Therefore, 279 patients were included in this analysis.

### Patient Demographics

The baseline characteristics are described in Table 1. The median age was 65 years (interquartile range [IQR], 59.0–70.5), and 223 (79.9%) were male. More than 96% of patients (n=268) were in CCS class II or III before randomization.

**Table 1.**
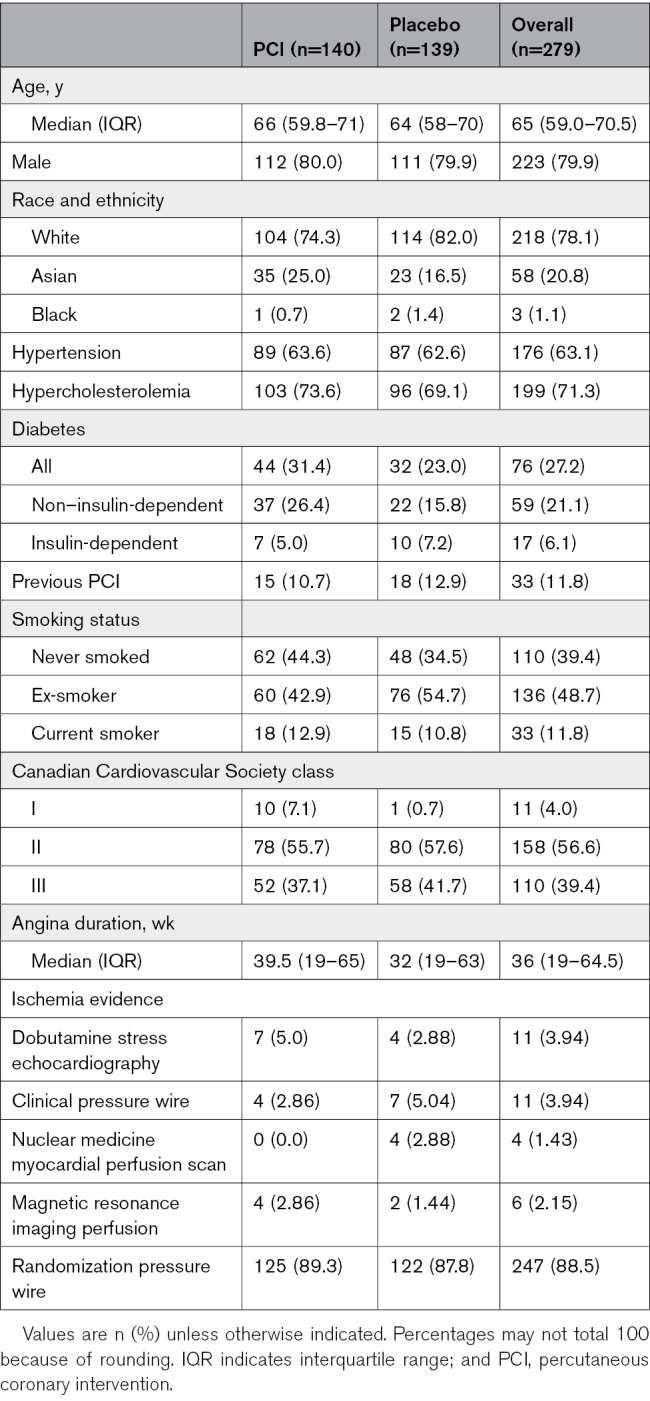
Baseline Characteristics

### Procedural Demographics

The procedural demographics are shown in Table 2; 224 (80.3%) patients had an angiographically severe stenosis in one coronary territory, 49 (17.6%) in 2, and 6 (2.2%) in all 3 coronary territories. The 279 patients had a total of 355 affected coronary arteries deemed suitable for treatment with PCI. The most frequently affected was the left anterior descending (197 of 355; 55.5%). The median FFR was 0.60 (IQR, 0.46–0.73), and iFR was 0.76 (IQR, 0.50–0.76). A total of 269 of 279 (96.4%) patients had an FFR ≤0.80, and 242 of 279 (86.7%) had an iFR ≤0.89. The median length of stent per patient in the PCI group was 30 mm (IQR, 21–49 mm). Postdilatation was performed in 217 of 252 stenoses (86.1%). In the PCI group, the median final FFR in the treated vessel was 0.88 (IQR, 0.84–0.93), and iFR was 0.93 (IQR, 0.91–0.97).

**Table 2.**
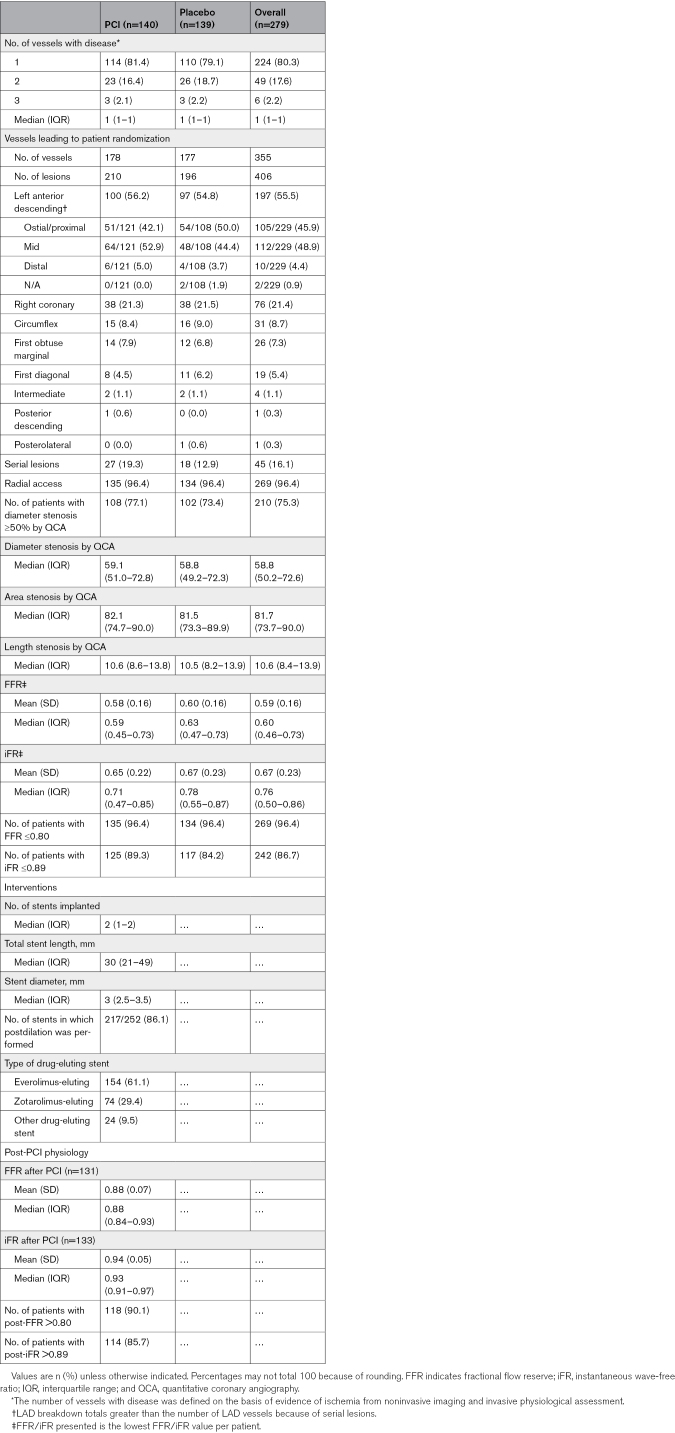
Procedural Characteristics

### Study End Points

#### Angina Symptom Score

**Figure 1.**
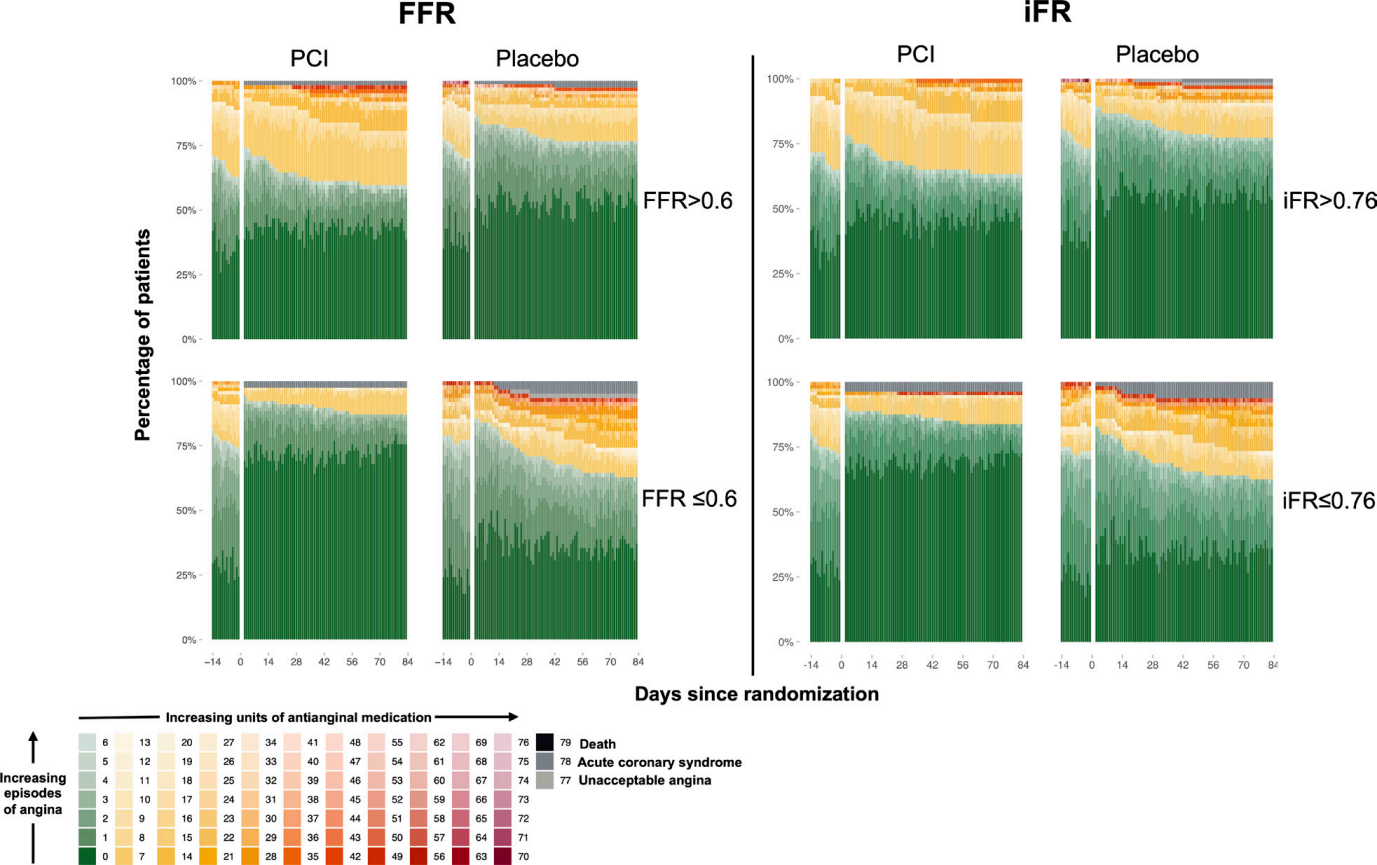
**The placebo-controlled effect of PCI on angina symptom score dichotomized by median FFR and iFR.** The angina symptom score ranges from 0 to 79, with lower scores indicating a better angina health status. It is calculated using the number of daily angina episodes, the number of units of anti-anginal medication prescribed that day, and high-level category override events (severe angina leading to unblinding, myocardial infarction, and death). FFR indicates fractional flow reserve; iFR, instantaneous wave-free ratio; and PCI, percutaneous coronary intervention.

**Figure 2.**
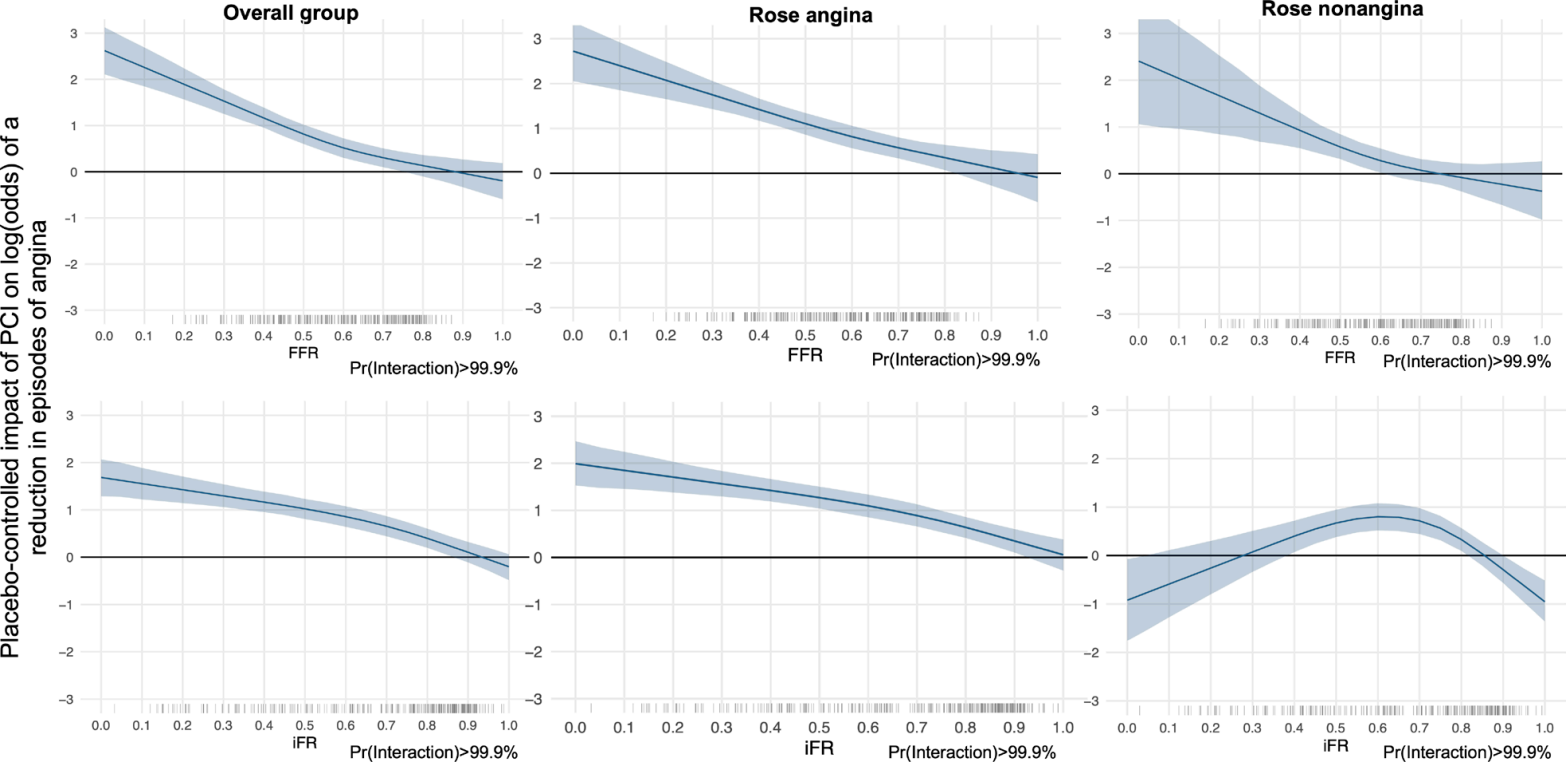
**Daily angina episodes stratified by FFR and iFR.** Vertical lines on the *x* axis represent the distribution of individual FFR and iFR values. Rose is a symptom characteristics questionnaire from which patients can be designated as having Rose angina or Rose nonangina. FFR indicates fractional flow reserve; iFR, instantaneous wave-free ratio; PCI, percutaneous coronary intervention; and Pr(Interaction), probability of interaction.

Angina symptom score data were available for 279 patients in the physiology-stratified analysis of ORBITA-2 (140 in the PCI group and 139 in the placebo group). There was strong evidence of benefit with PCI over placebo on angina symptom score in this group (OR, 1.82; 95% credible interval [CrI], 1.51–2.21; probability of benefit [Pr(Benefit)]>99.9%). A visualization of the daily angina symptom score data dichotomized using the median FFR and iFR values is shown in Figure 1. There was strong evidence of an interaction between both FFR and iFR, and the placebo-controlled impact of PCI on angina symptom score, with lower FFR and iFR values associated with greater treatment response (FFR OR, 2.01; 95% CrI, 1.79–2.26; probability of interaction [Pr(Interaction)]>99.9%; iFR OR, 2.13; 95% CrI, 1.87–2.45, Pr(Interaction)>99.9%; Tables 3 and 4; Figure 2).

**Table 3.**
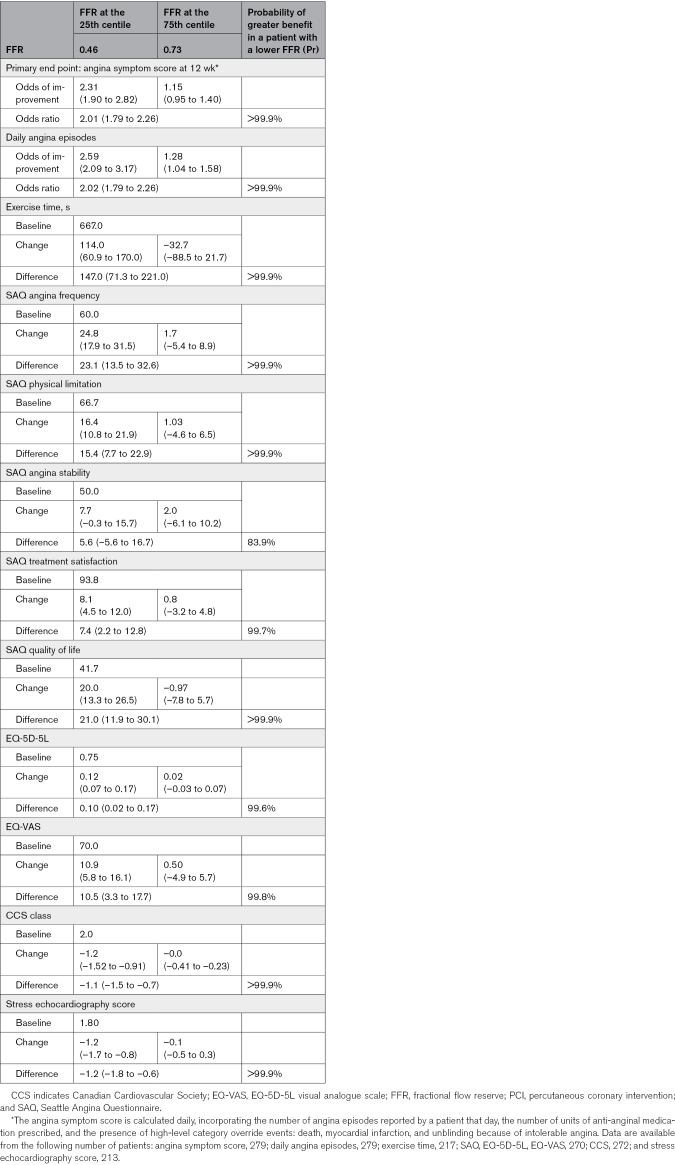
FFR Primary and Secondary End Points

**Table 4.**
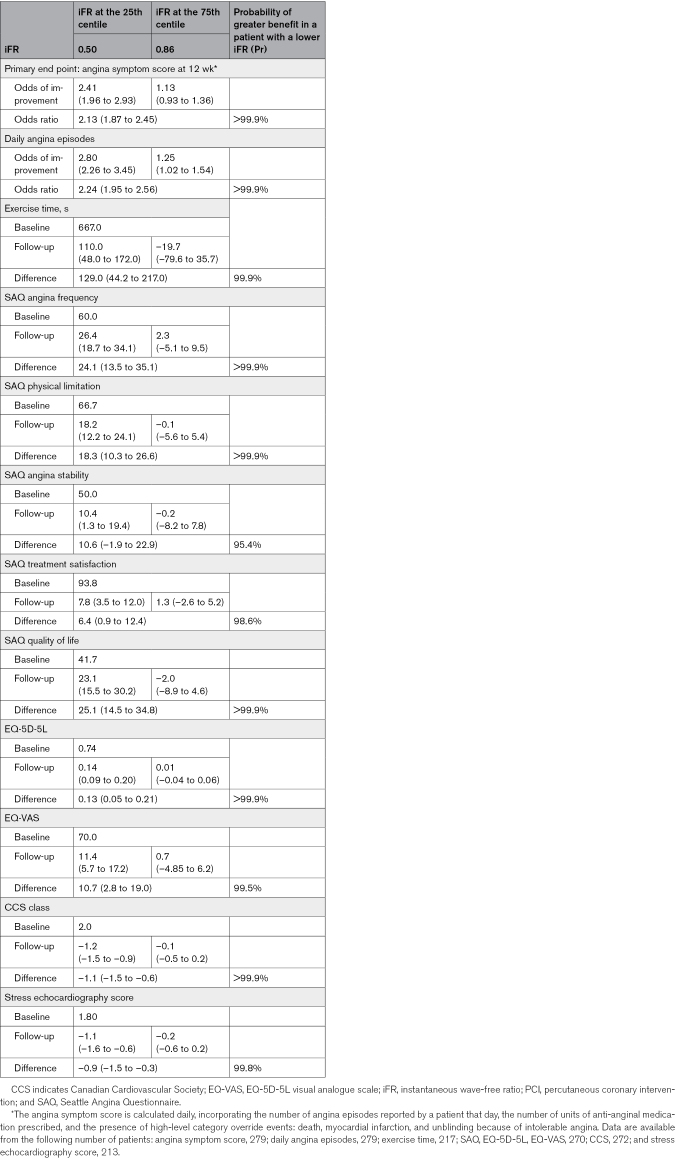
iFR Primary and Secondary End Points

#### Daily Angina Episodes

**Figure 3.**
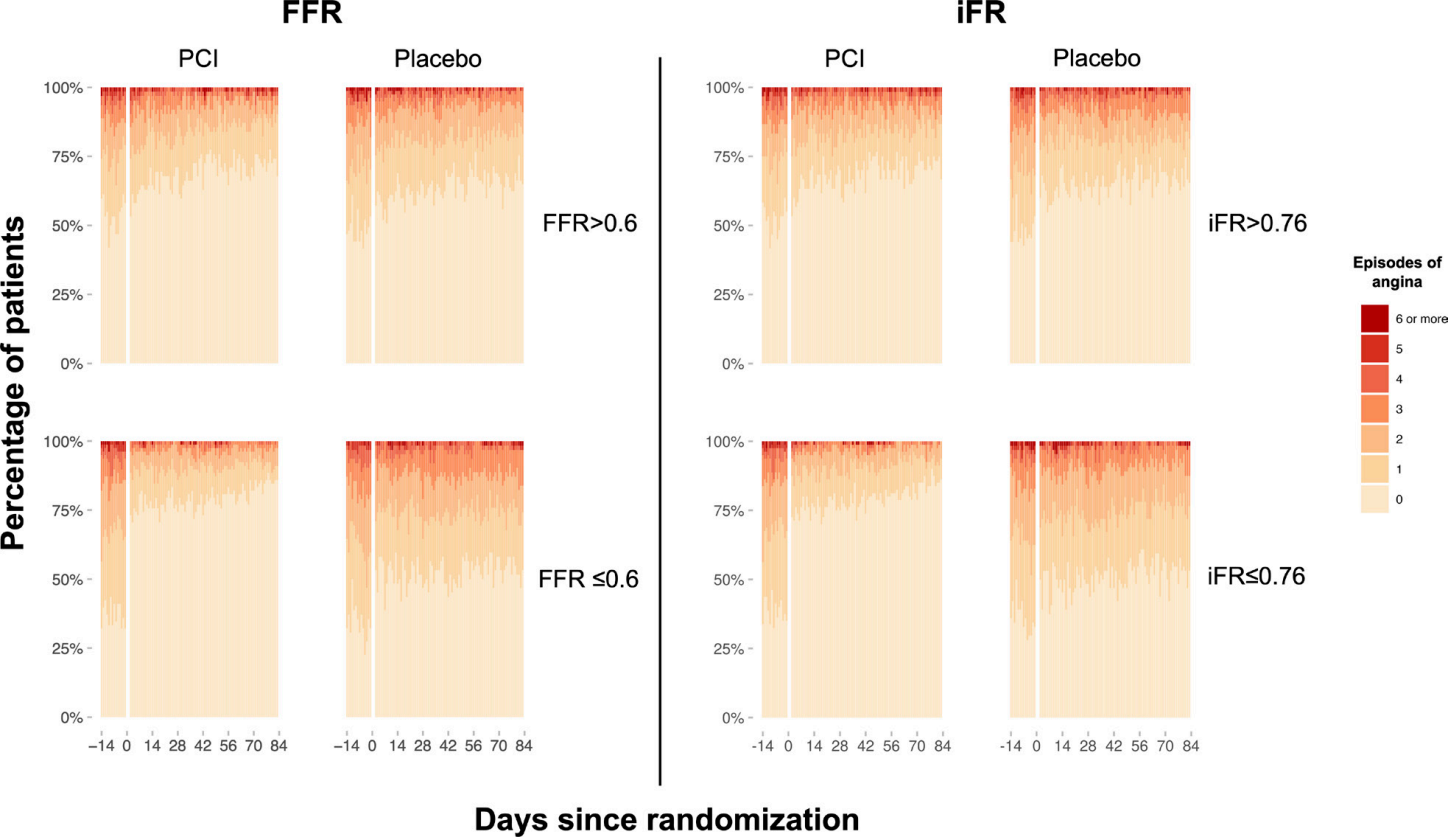
**The placebo-controlled effect of PCI on daily angina episodes dichotomized by median FFR and iFR.** FFR indicates fractional flow reserve; iFR, instantaneous wave-free ratio; and PCI, percutaneous coronary intervention.

Overall, there was strong evidence of benefit with PCI over placebo on daily angina episodes (OR, 1.84; 95% CrI, 1.51–2.24; Pr(Benefit)>99.9%) in this physiology subgroup. There was strong evidence of an interaction between both FFR and iFR and the placebo-controlled effect of PCI on the number of daily angina episodes (FFR OR, 2.02; 95% CrI, 1.79–2.26; Pr(Interaction)>99.9%; iFR OR, 2.24; 95% CrI, 1.95–2.56; Pr(Interaction)>99.9%; Tables 3 and 4; Figure 2). The daily angina episodes dichotomized using median FFR and iFR values are shown in Figure 3.

#### Patient-Reported and Physician-Assessed Symptoms and Quality of Life

Questionnaire data were available from 270 patients in this analysis (136 in the PCI group and 134 in the placebo group), and CCS data were available from 272 patients (137 in the PCI group and 135 in the placebo group).

**Figure 4.**
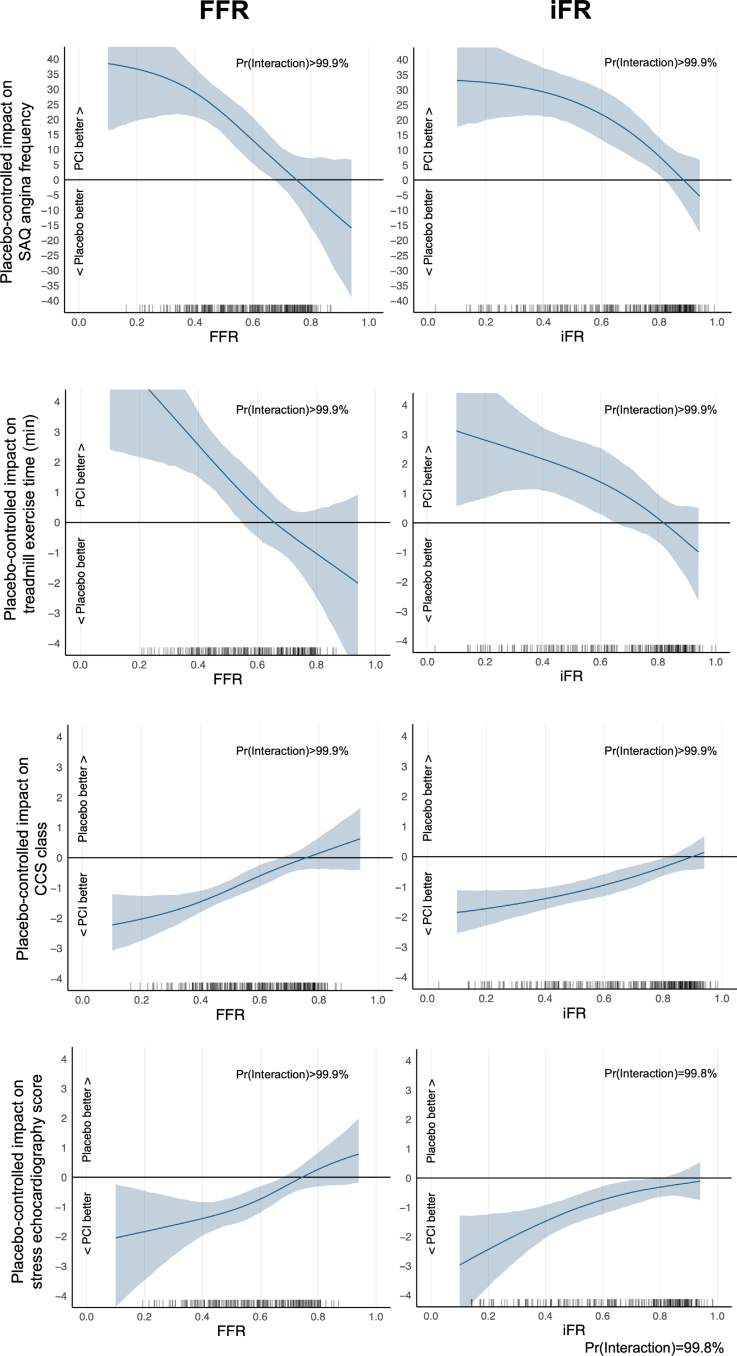
**Physiology-stratified placebo-controlled PCI treatment effect.** The placebo-controlled effect of PCI on SAQ angina frequency, CCS class, treadmill exercise time, and dobutamine stress echocardiography score stratified by FFR and iFR. Vertical lines on the *x* axis represent the distribution of individual FFR and iFR values. CCS indicates Canadian Cardiovascular Society; FFR, fractional flow reserve; iFR, instantaneous wave-free ratio; PCI, percutaneous coronary intervention; Pr(Interaction), probability of interaction; and SAQ, Seattle Angina Questionnaire.

There was strong evidence of benefit with PCI over placebo on SAQ angina frequency (14.1; 95% CrI, 9.1–19.4; Pr(Benefit)>99.9%). There was strong evidence of an interaction between both FFR and iFR and the placebo-controlled response to PCI on SAQ angina frequency (FFR, 23.1; 95% CrI, 13.5–32.6; Pr(Interaction)>99.9%; iFR, 24.1; 95% CrI, 13.5–35.1; Pr(Interaction)>99.9%; Tables 3 and 4; Figure 4). The interaction between FFR and iFR and the other components of the SAQ are in the Supplemental Material (Figures S22, S30, S38, S46, S101, S109, S117, and S125).

There was evidence of benefit with PCI over placebo in the EQ-5D-5L descriptive system (0.07; 95% CrI, 0.03–0.11; Pr(Benefit)>99.9%) and in the visual analogue scale (5.9; 95% CrI, 2.0–9.7; Pr(Benefit)=99.9%). There was strong evidence of interaction between both FFR and iFR and the impact of PCI on the EQ-5D-5L descriptive system and EQ VAS, with lower values associated with a larger treatment response (Tables 3 and 4).

PCI improved CCS class compared with placebo (–0.69; 95% CrI, –0.92 to –0.44; Pr(Benefit)>99.9%). There was strong evidence of interaction between FFR and iFR and placebo-controlled improvement in CCS class with PCI, with lower values associated with greater treatment response (FFR, –1.1; 95% CrI, –1.15 to –0.7; Pr(Interaction)>99.9%; iFR. –1.1; 95% CrI, –1.5 to –0.6; Pr(Interaction)>99.9%; Tables 3 and 4; Figure 4).

#### Treadmill Exercise Time

Treadmill exercise test data were available for 217 patients in the physiology-stratified analysis (114 in the PCI arm and 103 in the placebo arm). The effect of PCI over placebo on exercise time in these patients was 46.0 s (95% CrI, 5.6–88.3; Pr[Benefit]=98.6%). There was strong evidence of an interaction between both FFR and iFR and the placebo-controlled impact of PCI on exercise time (FFR, 147.0 s; 95% CrI, 71.3–221.0; Pr[Interaction]>99.9%; iFR, 129.0 s; 95% CrI, 44.2–217.0; Pr[Interaction]=99.9%; Tables 3 and 4; Figure 4), with lower values associated with greater treatment response.

#### Dobutamine Stress Echocardiography

DSE data were available from 213 patients in this analysis (110 in the PCI group and 103 in the placebo group). PCI caused a reduction in stress echocardiography score compared with placebo (–0.77; 95% CrI, –1.15 to –0.41; Pr[Benefit]>99.9%). There was strong evidence of an association between FFR and iFR and stress echocardiography score (FFR, –1.2; 95% CrI, –1.8 to –0.6; Pr[Benefit]>99.9%; iFR, –0.9; 95% CrI, –1.5 to –0.3; Pr[Benefit]=99.8%; Tables 3 and 4; Figure 4) with lower values associated with greater treatment response.

### Invasive Physiology and Symptom Characteristics

There was evidence of an interaction between both FFR and iFR and the effect of PCI on angina symptom score in patients with Rose angina (FFR OR, 2.05; 95% CrI, 1.75–2.41; Pr[Interaction]>99.9%; iFR OR, 2.11; 95% CrI, 1.79–2.53; Pr[Interaction]>99.9%). In patients with Rose nonangina, there was strong evidence of greater benefit with PCI at lower FFR and iFR values, but this relationship was weaker than in patients with Rose angina (FFR OR, 1.63; 95% CrI, 1.27–2.09; Pr[Interaction]>99.9%; iFR OR, 1.69; 95% CrI, 1.32–2.17; Pr[Interaction]>99.9%), with no evidence of benefit around the clinically used cut points (Figure 2). A similar association between symptom characteristics and the physiology-stratified effect of PCI was seen with all other end points (Figures S7, S8, S15, S23, S31, S39, S47, S54, S61, S69, S76, S94, S102, S110, S118, S126, S133, S141, S148, and S155).

## DISCUSSION

This physiology-stratified analysis of ORBITA-2 shows that FFR and iFR predict placebo-controlled angina reduction with PCI. For the first time, invasively measured vessel-specific indices have demonstrated an ability to predict the blinded symptom response to PCI. Furthermore, symptom characteristics influence the association between invasive coronary physiology and the efficacy of PCI. To see symptom benefit with PCI in patients with Rose nonangina, lesions needed to be more physiologically significant than in patients with Rose angina.

FFR and iFR are surrogate markers of myocardial ischemia and are frequently used to guide PCI in stable coronary artery disease, according to widely accepted binary clinical thresholds.^13,14^ These cut points are often thought of as indicators that first, the stenosis is causing angina, and second, treating the stenosis will relieve angina. However, although previous unblinded studies had suggested there may be an interaction between baseline FFR and angina response with PCI,^21^ these assumptions were not previously supported by blinded data.^5^

Symptom characteristics have already been found to be important predictors of the placebo-controlled efficacy of PCI.^12^ Patients with Rose angina are the most likely group to derive symptom benefit from PCI. This further analysis shows that in these patients, there is also a strong association between FFR and iFR and angina relief. However, even in patients with typical symptoms, the effect of PCI was more marginal and less certain with higher FFR and iFR values, particularly around the binary clinical thresholds. It is important to note that this relationship was also seen in patients with Rose nonangina; however, much lower invasive physiology values were needed to detect symptom benefit with PCI, and the effect size was diminished.

In contrast with this analysis of ORBITA-2, in ORBITA, no relationship was found between FFR and iFR and placebo-controlled angina improvement.^5^ Possible explanations for this include the different end points used in the respective trials, the inclusion of multivessel disease, and the additional statistical power provided by a larger sample size. Perhaps the most likely reason is the difference in anti-anginal regimens in the respective trials and the relationship between anti-anginal medications, angina, and ischemia. Patients were taking an average of 3 anti-anginals each in ORBITA, whereas in ORBITA-2, anti-anginal medications were stopped.^11,22^ Anti-anginal medications improve angina compared with placebo^7–9^ and have been shown to reduce myocardial ischemia in experimental settings.^10^ Perhaps the impact of a stenosis on angina and ischemia is attenuated by the presence of anti-anginal medication. The landmark association between FFR and noninvasive measures of ischemia was established in patients taking no anti-anginal medication.^23^ It may be that the ability of FFR and iFR to predict the effect of treating a stenosis and relieving angina and ischemia is less powerful in the presence of anti-anginal medications.

For many decades, we have focused on finding and eliminating myocardial ischemia on the assumption that this approach would lead to prognostic benefit. The use of anatomic and ischemic tests remains widespread in patients with a broad range of symptoms, particularly in the presence of cardiovascular risk factors. Therefore, in contemporary clinical practice, many patients with symptoms that may not be cardiac are referred to the cardiac catheterization laboratory. The physiology-stratified secondary analysis of ORBITA-2 can inform our management of these patients, helping to target PCI to those most likely to benefit and minimize risk to those with the least to gain. Now that the primary role of PCI in stable coronary artery disease is as an anti-anginal procedure, the relationship between symptom characteristics, physiology, and PCI treatment response should have a more prominent role in our clinical practice. Symptom assessment must take place before any anatomic or ischemic test. Patients with typical angina have the most to gain with PCI. In patients with atypical symptoms, the cause may be multifactorial and not solely attributable to the epicardial stenosis. Consequently, symptom improvement with PCI is seen only in those with the most physiologically severe stenoses (well below an FFR of 0.80 and an iFR of 0.89). This may be a setting in which invasive coronary physiology adds greater additional value.

### Limitations

Every patient in ORBITA-2 had evidence of myocardial ischemia. In 88.5% of patients, this evidence was from pressure wire assessment during the randomization procedure. Patients with no other evidence of ischemia who had FFR and iFR measurements above the clinically used thresholds were not randomized. Therefore, the majority had FFR and iFR measurements below the clinically used thresholds, and this secondary analysis does not represent the full physiological range.

After 12 weeks, patients were unblinded and returned to routine care. Therefore, the longer-term effect of PCI, and the interaction between FFR and iFR on this effect, remain uncertain.

In ORBITA-2, patients were treated with the minimum tolerated anti-anginal medication. The relationship between invasive physiology and treatment response may vary on higher levels of anti-anginal medication.

### Conclusions

Invasive physiology can be used to predict symptom response with PCI. It is particularly useful in patients with little or no concomitant anti-anginal therapy. The strength of this relationship is in part dependent on the nature of symptoms. This physiology-stratified analysis of ORBITA-2 provides a framework to integrate symptoms and invasive physiological assessment in the contemporary use of PCI as an anti-anginal procedure.

## ARTICLE INFORMATION

### Sources of Funding

ORBITA-2 was an investigator-initiated trial sponsored by Imperial College London. The trial was funded by grants from the National Institute for Health and Care Research Imperial Biomedical Research Centre, Medical Research Council, British Heart Foundation, National Institute for Health and Care Research, and the Imperial Coronary Flow Trust. Philips Volcano supplied the coronary pressure wires.

### Disclosures

Dr Foley has received speaker fees from Menarini and has received consulting and speaker fees from Shockwave Medical, Inc, and Philips. Dr Rajkumar has received speaker fees from Menarini, has received consulting fees from Philips, and has received shares in Mycardium AI. Dr Simader has received a sponsorship from Servier Pharmaceuticals. Dr Khokhar has received speaker fees and travel support from Boston Scientific and Abbott. Dr Davies has received grants from Medtronic and Abbott; has received sponsorship from Vascular Perspectives, Boston Scientific, Medtronic, and Abbott; and has received speaker fees from AstraZeneca, Pfizer, Bristol Myers Squibb, and Novartis. Dr Keeble has served on advisory boards for Abbott Vascular and SMT; and has received institutional research funding from Terumo, Medtronic, Boston Scientific, Abbott Vascular, Philips Volcano, and Cardionovum. Dr O’Kane has received speaker fees from Abbott Vascular, Biosensors, Boston Scientific, Heartflow, Medtronic, Philips, Shockwave, and Terumo. Dr Kotecha has received honoraria from Bayer and Janssen. Dr Nijjer has received speaker fees from Philips Volcano, Pfizer, Bayer, AstraZeneca, Boehringer Ingelheim, and Amarin. Dr Spratt has received speaker fees from Boston Scientific Corporation and Shockwave Medical, Inc. Dr Sen has received speaker and consulting fees from Philips, Medtronic, Recor, and AstraZeneca. Dr Curzen has received grants from Beckman Coulter, Inc, Boston Scientific Corporation, Haemonetics Corporation, and HeartFlow Inc; and has received speaker fees from Heartflow. Dr Howard has received shares in Mycardium AI; and has received a grant from the British Heart Foundation. Dr Cole has received shares in Mycardium AI. Dr Al-Lamee has served on advisory boards for Janssen Pharmaceuticals, Abbott, and Philips; and has received speaker fees from Abbott, Philips, Medtronic, Servier, Omniprex, and Menarini. The other authors report no conflicts.

### Supplemental Material

Supplemental Material

Tables S1–S7

Figures S1–S162
